# Application of 3D nnU-Net with Residual Encoder in the 2024 MICCAI Head and Neck Tumor Segmentation Challenge

**DOI:** 10.1007/978-3-031-83274-1_20

**Published:** 2025-03-03

**Authors:** Kaiyuan Ji, Zhihan Wu, Jing Han, Jun Jia, Guangtao Zhai, Jiannan Liu

**Affiliations:** 1 School of Communication and Electronic Engineering, East China Normal University, Shanghai, China; 2 Department of Oral and Maxillofacial Head and Neck Oncology, Shanghai Ninth People’s Hospital, Shanghai Jiao Tong University School of Medicine, Shanghai, China; 3 School of Electronic Information and Electrical Engineering, Shanghai Jiao Tong University, Shanghai, China

**Keywords:** Artificial Intelligence, Deep Learning, Medical Image, Tumor Segmentation

## Abstract

This article explores the potential of deep learning technologies for the automated identification and delineation of primary tumor volumes (GTVp) and metastatic lymph nodes (GTVn) in radiation therapy planning, specifically using MRI data. Utilizing the high-quality dataset provided by the 2024 MICCAI Head and Neck Tumor Segmentation Challenge, this study employs the 3DnnU-Net model for automatic tumor segmentation. Our experiments revealed that the model performs poorly with high background ratios, which prompted a retraining with selected data of specific background ratios to improve segmentation performance. The results demonstrate that the model performs well on data with low background ratios, but optimization is still needed for high background ratios. Additionally, the model shows better performance in segmenting GTVn compared to GTVp, with DSCagg scores of 0.6381 and 0.8064 for Task 1 and Task 2, respectively, during the final test phase. Future work will focus on optimizing the model and adjusting the network architecture, aiming to enhance the segmentation of GTVp while maintaining the effectiveness of GTVn segmentation to increase accuracy and reliability in clinical applications.

## 1 Introduction

In the field of oncology, the assessment and treatment of primary gross tumor volumes (GTVp) and metastatic mymph dodes (GTVn) hold significant importance in clinical decision-making and research [1]. Traditionally, in radiation therapy planning, physicians were required to manually identify and delineate tumors and affected lymph nodes, a process that was not only time-consuming but also susceptible to subjective biases from individual physicians [2, 3]. In recent years, with the advancement of artificial intelligence technologies, deep learning has offered substantial potential in the field of medical image segmentation [4, 5]. By training models to recognize GTVp and GTVn in medical images, the accuracy of segmentation has been further enhanced [6]. These models learn a variety of visual features of tumors, including shape, size, and contrast with surrounding tissues, through extensive training data [7, 8]. Once trained, these models are capable of automatically processing new images, swiftly identifying tumor regions and affected lymph nodes, significantly accelerating the workflow for treatment planning [9, 10].

The annual Medical Image Computing and Computer Assisted Intervention Society (MICCAI) HEAD AND NECK TUMOR SEGMENTATION FOR MR-GUIDED: APPLICATIONS (HNTS-MRG) 2024 Challenge provided a series of high-quality, annotated T2-weighted datasets of patients who underwent radiotherapy (RT), including pre-RT (1–3 weeks before the start of RT) and mid-RT (2–4 weeks intra-RT) [11]. This offered a pathway for the systematic evaluation of automated segmentation methods for GTVp and GTVn. The study primarily utilized a residual encoder version of 3DnnU-Net to perform tumor segmentation for both the pre and mid-RT tasks.

## 2 Methods

T2w MRI imaging data from head and neck cancer patients who underwent radiotherapy (Sect. 2.1) was used to develope deep learning models for auto-segmentation of tumors and multiple lymph nodes. The ground truth manual segmentations and the classified imaging data (Sect. 2.2) were uesd to train our models (Sect. 2.3).

### 2.1 Imaging Data

This study utilized the training dataset from the MICCAI 2024 Head and Neck Tumor Segmentation for MR-Guided Applications (HNTSMRG) 2024 Challenge [11]. The dataset consists of 150 patients with histologically proven head and neck cancer who underwent radiotherapy (RT) at The University of Texas MD Anderson Cancer Center. For each patient, the dataset includes a pre-RT T2w MRI scan (1–3 weeks before start of RT) and a mid-RT T2w MRI scan (2–4 weeks intra-RT). Images consist of a mix of fat-suppressed and non-fat-suppressed MRI sequences.

All imaging data in the training set (from 150 patients) were manually segmented by multiple physician experts, and the segmentations were combined using the STAPLE (Simultaneous Truth and Performance Level Estimation) algorithm to generate the final ground truth segmentation labels. Both training and testing data were provided in Neuroimaging Informatics Technology Initiative (NIfTI) format.

### 2.2 Data Processing

All images were cropped from the top of the clavicles to the bottom of the nasal septum (oropharynx region to shoulders) by HNTSMRG2024 Challenge organizers to ensure consistency in the field of view and to remove identifiable facial structures. We divided the dataset into two groups based on the proportion of background present in the MRI images: 1) a group with 70%–90% background, containing 60 cases; and 2) a group with less than 70% background, containing 90 cases. This classification was made to explore the potential impact of different background ratios on model performance. To further enhance the smoothness of the segmentation mask boundaries, we employed a smoothing algorithm using 3D Slicer software [12]. This preprocessing step was applied to the manually annotated training data to refine the segmentation masks by reducing isolated segmented regions and smoothing the boundaries. The improved segmentation contributes to the robustness and accuracy of the model during training.

In addition, we applied various data augmentation techniques to further improve the generalization capability of the model. These techniques included random rotations, scaling, and mirroring (horizontal and vertical) of images and their corresponding labels. We also introduced Gaussian noise and Gaussian blur to enhance the model’s robustness to noise and varying image quality. Adjustments to brightness and contrast were applied randomly to simulate diverse lighting conditions. To mimic low-resolution images, we utilized average pooling for downsampling, simulating a lower resolution. Gamma correction with random gamma values was used to perform nonlinear grayscale mapping. These augmentations were applied dynamically during training using nnU-Net, which randomly combines them as a foundational and general data augmentation strategy to enrich the training dataset and boost model performance.

### 2.3 Model Architecture

The nnU-Net residual architecture for 3D medical imaging processes data with a detailed and structured approach. Initially, it normalizes the images using Z-Score normalization and resamples the data and segmentation masks to achieve uniform voxel spacing and dimensions. It handles a patch size of 48 × 192 × 192 voxels, with spacing configured to 1.2 mm × 0.5 mm × 0.5 mm. The architecture employs 3D convolution operations with 3 × 3 × 3 kernels and utilizes strides such as [1, 2] and [2] to progressively downsample feature maps, simultaneously increasing the depth and number of features, which start at 32 and rise to 320 in deeper stages. It incorporates 1 to 6 residual blocks per stage, featuring instance normalization and LeakyReLU activation to maintain non-linearity and computational efficiency. Overall, this complex setup with six stages is designed to capture a comprehensive range of features from medical images, facilitating accurate segmentation tasks through a deep learning network. For more detailed information and specifics, refer to the nnU-Net’s residual encoder-generated plans.json file. For more detailed information, refer to the plans.json file generated after using the nnU-Net residual architecture code.

### 2.4 Model Training Approach

In the training phase, we initially conducted mixed training with 450 samples containing pre, mid, and pre-registered images, completing a total of 1600 iterations. The learning rate was initialized at 0.01 and gradually decreased to 0 by the end of training. We used an SGD optimizer with a weight decay of 3 × 10^−5^, and the batch size was set to 2. Additionally, we employed 5-fold cross-validation to ensure robust evaluation. Dice and cross-entropy were used as loss functions to guide the optimization process.

During the subsequent testing phase, we observed that the model performed poorly on certain parts of the dataset. Analysis revealed that this issue was primarily caused by a high proportion of background (labeled as 0) in the dataset. Notably, in these imaging data, the proportion of background ranged from approximately 4% to 90%.

To more effectively segment data with a higher background ratio, we selected samples where the background constituted about 70% to 90%, forming a separate dataset for training. Similarly, we included pre-registered data with a background ratio of 50% to 80% in the training. Both of these additional training phases underwent 500 iterations. For mid images, we adopted a similar strategy, selecting samples with a background ratio of 70% to 90% for independent training, which also involved 500 iterations. Importantly, during these subsequent training phases, all hyperparameters, including the learning rate schedule, optimizer (SGD with a weight decay of 3 × 10^−5^), batch size (2), and loss functions (Dice and cross-entropy), were kept consistent. The only difference was the number of iterations, which was adjusted based on the dataset.

**Fig. 1.**
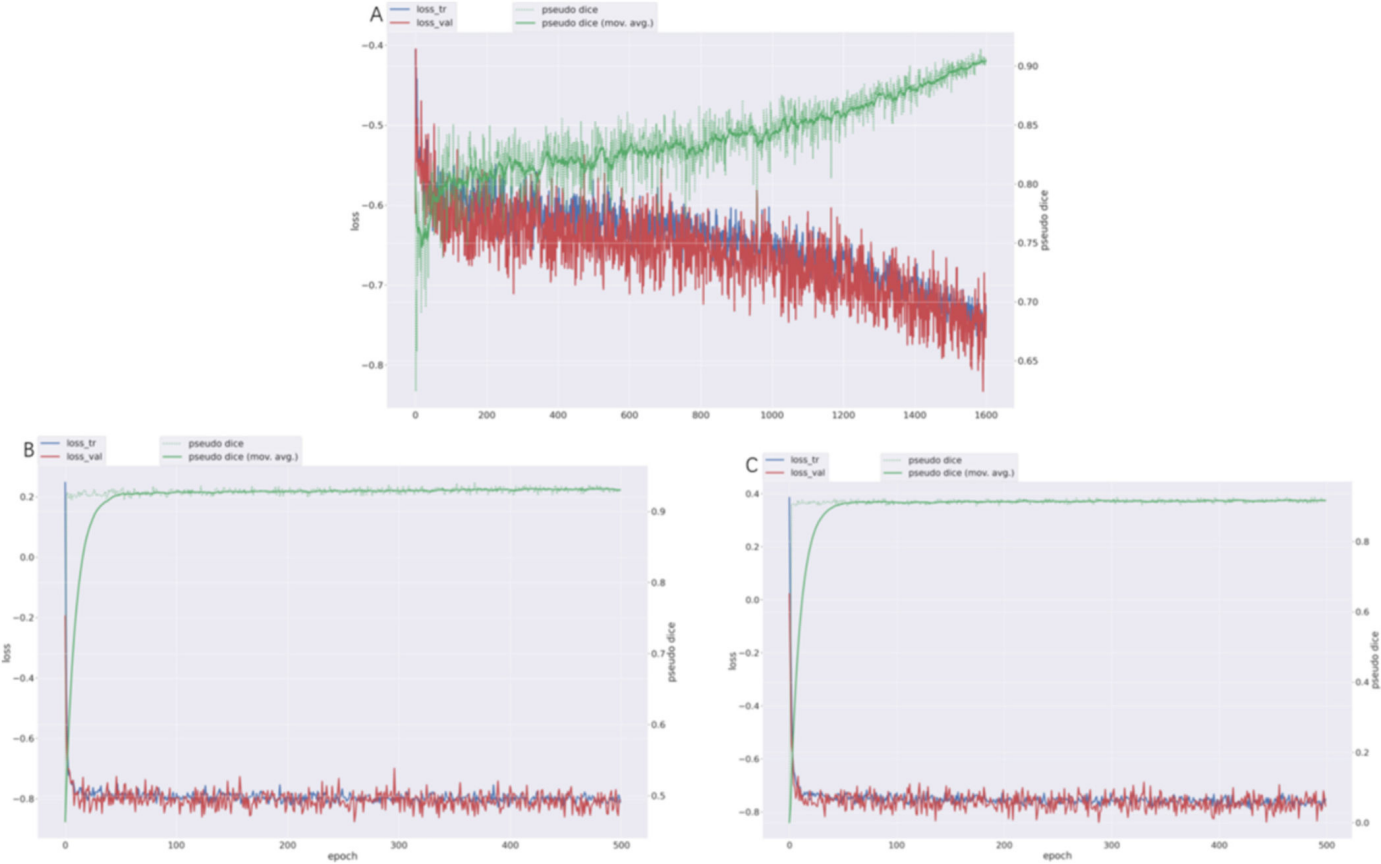
Training curves for three models (A) Training curve for the mixed model (B) Training curve for the Pre-RT segmentation model with a high background ratio (C) Training curve for the Mid-RT segmentation model.

The associated training curves are shown in Fig. 1. The visualization metric used here was the Dice coefficient, rather than the DSCagg (aggregated Dice Similarity Coefficient) used by the official competition. The Dice coefficient served only as a display, with final results based on DSCagg. Through stratified training, we discovered that segmentation accuracy improved when the model was specifically trained on images with higher background ratios, suggesting that adapting the training process to the characteristics of the dataset, such as background proportions influenced by the field of view and underlying tumor volumes, can enhance model performance. Additionally, considering the inherent variations in imaging quality, such as resolution indicated by background ratio, we found that training dedicated models for these specific data distributions yielded better results. A simple cropping operation, while effective in reducing unnecessary elements, was evaluated but ultimately deemed less critical than focused training, as the trained model tends to ignore large zero-value areas and concentrate on regions with pixel activity. For images with a high background ratio, after analysis by the model, train the model using preprocessing parameters different from those used for images with a low background ratio, so that it focuses solely on their internal distributions, thereby enhancing the segmentation capabilities of the model to a certain extent.

## 3 Results

**Fig. 2.**
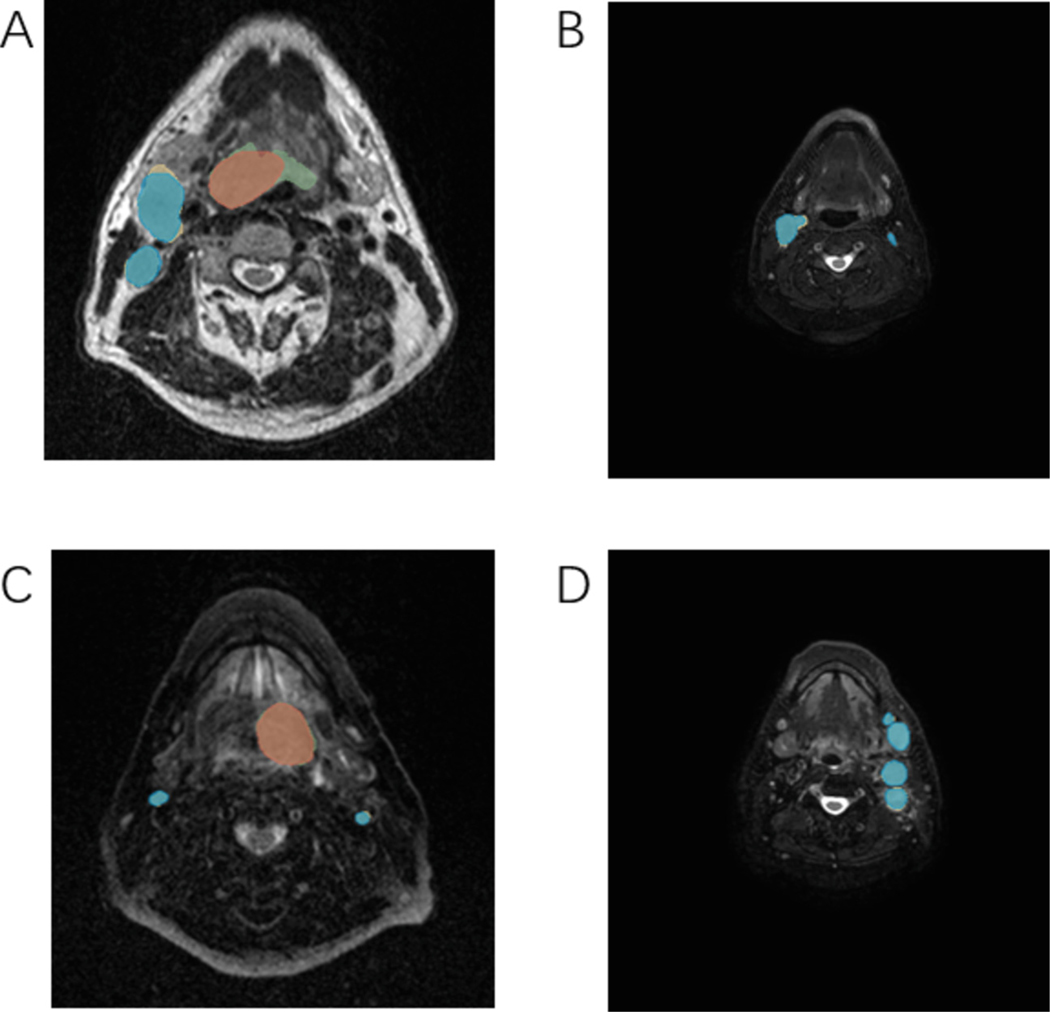
Head tumor segmentation results for Pre-RT and Mid-RT segmentation. Green and yellow represent the annotation results for GTVp and GTVn, respectively, while red and blue represent the segmentation results for GTVp and GTVn, respectively. (A) Pre-RT tumor segmentation with low background proportion. (B) Pre-RT tumor segmentation with high background proportion. (C) Mid-RT tumor segmentation with low background proportion. (D) Mid-RT tumor segmentation with high background proportion.

Figure 2 shows the segmentation example results from our study. It is evident that for imaging data with a lower background proportion, the segmentation performance is quite satisfactory, effectively extracting the target areas with high accuracy and robustness. This indicates that our model performs well in scenarios with minimal distractions, accomplishing the segmentation tasks effectively. However, for imaging data with a higher background proportion, the results are less than ideal, possibly due to interference from background information, which complicates the model’s ability to distinguish between target and background [13]. We believe further optimization of the model is necessary to enhance its performance in complex background situations. Additionally, Table 1 presents the results from the test set after our competition submission. It is observed that, compared to the segmentation of GTVp, our model demonstrates superior performance in handling the segmentation tasks for GTVn. Specifically, the mean DSCagg values, which were used in the actual challenge ranking, are 0.8380 for GTVn (combining 0.8453 from one test subset and 0.8307 from another) and 0.6065 for GTVp (combining 0.7674 from one test subset and 0.4456 from another). These results underline our model’s enhanced capability in accurately segmenting metastatic lymph nodes compared to primary tumor volumes.

## 4 Discussion

In this study, we explored the impact of varying background ratios on the performance of segmentation models. By training with a mix of 450 samples, we initially established a baseline model. However, during the testing phase, we observed that the model performed poorly on images with higher background ratios. This phenomenon highlighted the importance of dataset distribution balance in model training [14, 15].

To address this issue, we implemented targeted measures by selecting samples with higher background ratios from the dataset to construct a new training set [16]. By concentrating the training on these samples, we aimed to enhance the model’s ability to segment complex backgrounds. This strategy somewhat mitigated the impact of background noise on segmentation effectiveness, yet it is important to note that the model might still struggle with very high background ratios.

Compared to the segmentation of GTVp, our model demonstrated better performance in segmenting GTVn. This could be due to the more distinct features of GTVn in images, or because the model learned the characteristics of GTVn more thoroughly. This difference indicates that, although the model is generally stable, there is still room for optimization when processing different types of tumor volumes. Understanding these subtle differences will help us further adjust and optimize the algorithm to enhance the model’s applicability and accuracy in various clinical scenarios [17, 18].

In future research, we plan to continue optimizing the model structure and explore using more data augmentation techniques and regularization methods to improve the model’s generalization ability in complex backgrounds [19]. Additionally, we hope to introduce more representative samples to better adapt the model to varying imaging data distributions, ultimately achieving more ideal segmentation results. Through ongoing experiments and improvements, we look forward to making significant progress in the field of medical image segmentation and providing more reliable technological support for clinical applications [20, 21].

Our study still faces limitations, as we have not improved the model to enhance the performance of GTVp. Additionally, we have not fully utilized the information from the other four inputs in the Mid-RT segmentation task.

## 5 Conclusion

This study employed 3D nnU-Net with a residual encoder, focusing on the complex task of head and neck tumor segmentation. We utilized meticulous data preprocessing and a multi-model training strategy to cater to various clinical segmentation needs. Specifically, according to Table 2, we achieved an excellent performance in the Pre-RT segmentation task with a DSCagg mean value of 0.8064, while the Mid-RT segmentation task yielded a mean DSCagg of 0.6381, indicating room for improvement.

Moreover, an analysis of the specific GTVp label performance in these two tasks, and their mean values, clearly highlighted the deficiencies in the model’s segmentation capabilities for this label. Future efforts were planned to focus on optimizing algorithms and adjusting the network structure, particularly improvements in GTVp segmentation, to enhance model accuracy and reliability in complex clinical applications. These improvements were not only expected to increase the precision of tumor monitoring before and after treatment but were also anticipated to provide more accurate treatment planning support in clinical settings.

## Figures and Tables

**Table 1.** Test set results for ensemble models. Metrics are reported from the HNTS-MRG 2024 submission portal.

Task	Tumor	DSCagg
Pre-RT segmentation	GTVn	0.8453
	GTVp	0.7674
Mid-RT segmentation	GTVn	0.8307
	GTVp	0.4456
